# Supplementary material for: Litter Management Strategies and Their Impact on the Environmental and Respiratory Microbiome Might Influence Health in Poultry
Source: Microorganisms. 2022 Apr 22;10(5):878. doi: 10.3390/microorganisms10050878 (PMC9144224; doi:10.3390/microorganisms10050878)

Table S1: Vaccines and Immunization Schedule.

Immunization Schedule			
Age of Administration	Name of Vaccine	Disease	Route of Administration
18-day-old chicken embryos	Innovax ND-IBD	Infectious bursal disease, Newcastle disease and Marek's disease	in ovo
1-day-old chicks	Nobilis IB Ma5	Infectious bronchitis	spray
1-day-old chicks	Nobilis IB 4-91	Infectious bronchitis	spray

Table S2: Number of birds used in this study and study groups distribution..

Birds used in this study				
	Week 1	Week 3	Week 6	Total
BH-I	3 birds	3 birds	3 birds	9
BH-II	3 birds	3 birds	3 birds	9
AF	3 birds	3 birds	3 birds	9
Total	9	9	9	27

AF: experimental animal facility; BH: broiler house.

Table S3: Tracheal sequences, OTUs and OPUs.

Flock	Time	Sample	Nr total sequences	Nr filtered sequences	*% filtered sequences*	Nr OTUs	Nr OPUS
BH-I	W1	1	12668	10644	*84.02*	65	26
BH-I	W1	2	10965	10930	*99.68*	112	58
BH-I	W1	3	14011	13988	*99.84*	28	8
		**Mean**	**12548**	**11854**	**95**	**68**	**31**
		**SD**	**1527**	**1854**	**9**	**42**	**25**
BH-II	W1	1	21322	16322	*76.55*	97	44
BH-II	W1	2	11339	11294	*99.60*	76	15
BH-II	W1	3	30251	30208	*99.86*	58	25
		**Mean**	**20971**	**19275**	**92**	**77**	**28**
		**SD**	**9461**	**9797**	**13**	**20**	**15**
AF	W1	1	20681	6849	*33.12*	77	46
AF	W1	2	26852	26777	*99.72*	110	53
AF	W1	3	13925	12573	*90.29*	71	29
		**Mean**	**20486**	**15400**	**74**	**86**	**43**
		**SD**	**6466**	**10260**	**36**	**21**	**12**
BH-I	W3	1	32127	32127	*100.00*	27	9
BH-I	W3	2	13670	13670	*100.00*	15	2
BH-I	W3	3	30417	30415	*99.99*	19	2
		**Mean**	**25405**	**25404**	**100**	**20**	**4**
		**SD**	**10198**	**10198**	**0**	**6**	**4**
BH-II	W3	1	10484	9998	*95.36*	26	9
BH-II	W3	3	12786	12783	*99.98*	16	3
		**Mean**	**11635**	**11391**	**98**	**21**	**6**
		**SD**	**1628**	**1969**	**3**	**7**	**4**
AF	W3	1	7323	2120	*28.95*	16	13
AF	W3	2	19684	14842	*75.40*	45	14
AF	W3	3	33650	17043	*50.65*	55	26
		**Mean**	**20219**	**11335**	**52**	**39**	**18**
		**SD**	**13172**	**8056**	**23**	**20**	**7**
BH-I	W6	1	14719	13133	*89.22*	65	35
BH-I	W6	2	19312	19190	*99.37*	86	32
BH-I	W6	3	18152	17344	*95.55*	64	22
		**Mean**	**17394**	**16556**	**95**	**72**	**30**
		**SD**	**2388**	**3104**	**5**	**12**	**7**
BH-II	W6	1	6577	6553	*99.64*	67	36
BH-II	W6	2	17848	17691	*99.12*	365	150
BH-II	W6	3	9635	9627	*99.92*	56	27
		**Mean**	**11353**	**11290**	**100**	**163**	**71**
		**SD**	**5829**	**5752**	**0**	**175**	**69**
AF	W6	1	12007	11614	*96.73*	83	58
AF	W6	3	26179	26167	*99.95*	57	18
		**Mean**	**19093**	**18891**	**98**	**70**	**38**
		**SD**	**10021**	**10291**	**2**	**18**	**28**
**Total**			446584	393902		856	281
**Mean**			17863	15756	88.50	70	30
**SD**			7997	7861	20.74	68	30

Figure S1: Number of tracheal sequences. Number of prokaryotic sequences obtained from tracheal wash samples (*n*=3). AF: experimental animal facility, BH: broiler house.

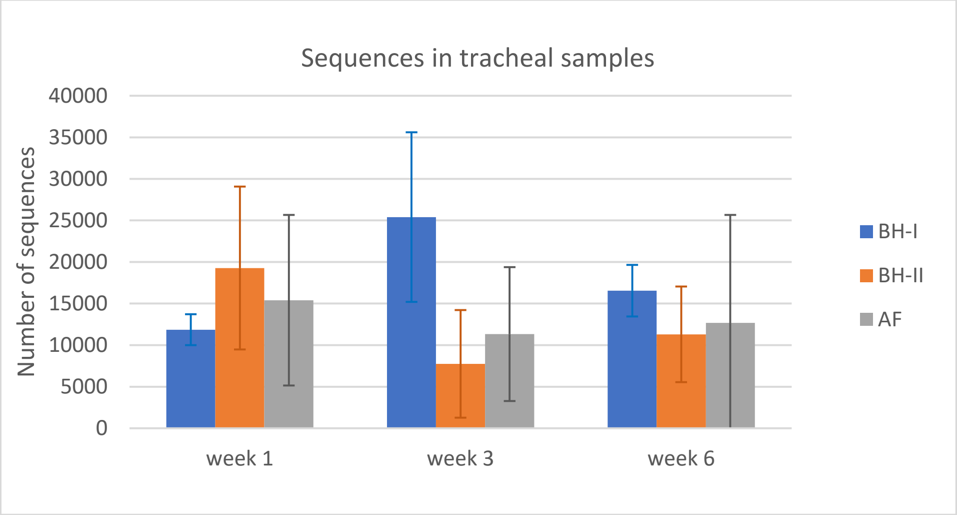

Table S4: Environmental air sequences, OTUs and OPUs.

Flock	Time	Nr total sequences	Nr filtered sequences	*% filtered sequences*	Nr OTUs	Nr OPUS
BH-I	W1	10926	10923	*99.97*	492	151
BH-II	W1	8500	8494	*99.93*	446	129
AF	W1	16503	16498	*99.97*	421	142
BH-I	W3	35365	35361	*99.99*	816	262
BH-II	W3	11832	11832	*100.00*	458	173
AF	W3	11612	11604	*99.93*	576	252
BH-I	W6	20303	20300	*99.99*	849	279
BH-II	W6	8492	8489	*99.96*	624	215
AF	W6	14916	14906	*99.93*	429	154
**TOTAL**		138449	138407		1527	486
**Mean**		15383	15379	99.96	568	195
**SD**		8415	8415	0.027	165	58

Figure S2: Number of environmental air sequences. Number of prokaryotic sequences after sampling 1000 liters of air in nitrocellulose membrane over PCA plates (*n=1*). AF: experimental animal facility, BH: broiler house.

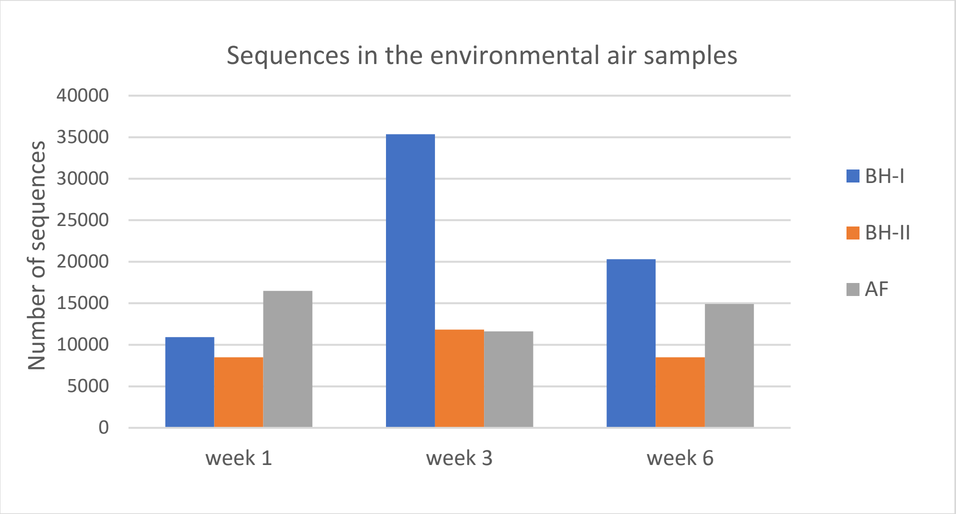

Table S5: Alpha diversity indices for environmental samples.

Flock	Time	Dominance-D index	Shannon–Weiner index	*Chao-1*
BH-I	W1	0.070	3.230	170
BH-II	W1	0.124	2.863	158
AF	W1	0.128	2.970	148
BH-I	W3	0.098	3.154	279
BH-II	W3	0.146	2.598	221
AF	W3	0.140	3.360	254
BH-I	W6	0.074	3.498	338
BH-II	W6	0.066	3.490	250
AF	W6	0.249	2.436	159

Table S6: Alpha diversity indices for tracheal samples.

Flock	Time	Sample	Dominance-D index	Shannon–Weiner index	*Chao-1*
BH-I	W1	1	0.118	2.563	27
BH-I	W1	2	0.617	1.189	61
BH-I	W1	3	0.848	0.381	8
		**Mean**	**0.527**	**1.378**	**32**
		**SD**	**0.373**	**1.103**	**27**
BH-II	W1	1	0.130	2.690	51
BH-II	W1	2	0.352	1.435	15
BH-II	W1	3	0.665	0.836	25
		**Mean**	**0.382**	**1.654**	**30**
		**SD**	**0.268**	**0.946**	**19**
AF	W1	1	0.063	3.086	56
AF	W1	2	0.653	1.029	57
AF	W1	3	0.108	2.532	30
		**Mean**	**0.274**	**2.216**	**47**
		**SD**	**0.328**	**1.064**	**16**
BH-I	W3	1	0.991	0.032	19
BH-I	W3	2	1.000	0.002	2
BH-I	W3	3	1.000	0.002	2
		**Mean**	**0.997**	**0.012**	**8**
		**SD**	**0.005**	**0.017**	**10**
BH-II	W3	1	0.963	0.125	9
BH-II	W3	3	0.987	0.042	3
		**Mean**	**0.975**	**0.084**	**6**
		**SD**	**0.017**	**0.059**	**4**
AF	W3	1	0.200	1.923	13
AF	W3	2	0.437	1.027	15
AF	W3	3	0.243	1.747	28
		**Mean**	**0.293**	**1.566**	**18**
		**SD**	**0.126**	**0.475**	**8**
BH-I	W6	1	0.230	2.149	35
BH-I	W6	2	0.269	1.659	50
BH-I	W6	3	0.858	0.340	26
		**Mean**	**0.452**	**1.383**	**37**
		**SD**	**0.352**	**0.936**	**12**
BH-II	W6	1	0.678	0.843	36
BH-II	W6	2	0.100	3.406	153
BH-II	W6	3	0.791	0.598	28
		**Mean**	**0.523**	**1.616**	**72**
		**SD**	**0.371**	**1.555**	**70**
AF	W6	1	0.161	2.592	63
AF	W6	3	0.462	1.131	20
		**Mean**	**0.312**	**1.862**	**41**
		**SD**	**0.213**	**1.033**	**31**

Table S7: Phylum and Class relative abundances for air environmental samples.

Phylum	BH-I_W1	BH-II_W1	AF_W1	BH-I_W3	BH-II_W3	AF_W3	BH-I_W6	BH-II_W6	AF_W6
Actinomycetota	0.29	2.04	0.64	38.01	38.62	4.88	27.60	25.96	1.41
Bacteroidota	0.03	0.00	0.79	1.20	0.30	0.67	0.71	0.15	0.64
Campylobacterota	0.00	0.00	0.00	0.01	0.00	0.00	0.10	0.07	0.00
Cyanobacteria	0.00	0.00	0.00	0.01	0.00	0.00	0.03	0.00	0.00
Bacillota	96.59	95.85	86.39	59.76	60.59	82.85	69.81	73.06	84.46
Pseudomonadota	3.10	2.10	12.18	1.02	0.49	11.61	1.74	0.75	13.49
Class	BH-I_W1	BH-II_W1	AF_W1	BH-I_W3	BH-II_W3	AF_W3	BH-I_W6	BH-II_W6	AF_W6
Acidimicrobiia	0.00	0.00	0.00	0.00	0.00	0.07	0.00	0.00	0.02
Actinobacteria	0.29	2.04	0.34	37.99	38.60	3.51	27.38	25.95	0.20
Alphaproteobacteria	0.00	0.00	0.20	0.00	0.00	0.06	0.00	0.00	0.01
Bacilli	81.58	84.08	70.93	58.04	59.39	68.47	63.81	66.58	71.79
Bacteroidia	0.03	0.00	0.79	1.20	0.30	0.67	0.71	0.15	0.64
Campylobacteria	0.00	0.00	0.00	0.01	0.00	0.00	0.10	0.07	0.00
Clostridia	15.00	11.77	15.46	1.71	1.20	14.38	6.01	6.48	12.67
Coriobacteriia	0.00	0.00	0.29	0.01	0.02	1.30	0.22	0.01	1.19
Gammaproteobacteria	3.10	2.10	11.98	1.02	0.49	11.55	1.74	0.75	13.48
Vampirivibrionia	0.00	0.00	0.00	0.01	0.00	0.00	0.03	0.00	0.00

Table S8: List of OPUs and their closest relative sequence .

OPU	Identification	Closest relative sequence / Sequence entry number
OPU-001	*Lactobacillaceae* sp.	*Lactobacillus acidophilus* / KC936149
OPU-005	*Lactobacillaceae* sp.	*Lactobacillus gasseri* / KY658480
OPU-006	*Limosilactobacillus* sp.	*Lactobacillus reuteri* / MF444688
OPU-019	*Ligilactobacillus* sp.	*Lactobacillus salivarius* / KC561118
OPU-026	*Leuconostoc mesenteroides* subsp. *mesenteroides* ATCC 8293	*Leuconostoc mesenteroides* subsp. *mesenteroides* ATCC 8293 / CP000414
OPU-030	*Streptococcus alactolyticus*	*Streptococcus alactolyticus* / AF201899
OPU-032	*Streptococcus* sp.	*Streptococcus pluranimalium* / CP022601
OPU-036	*Enterococcus* sp.	*Enterococcus hirae* / KC707583
OPU-042	*Enterococcus* sp.	*Enterococcus columbae* / AF061006
OPU-059	*Aerococcus* sp.	*Aerococcus suis* / AM230658
OPU-104	*Staphylococcus* sp.	*Staphylococcus saprophyticus* subsp. *saprophyticus* ATCC 15305 / AP008934
OPU-108	*Staphylococcus* sp.	*Staphylococcus equorum* / CP013114
OPU-110	*Staphylococcus* sp.	*Staphylococcus arlettae* / AB009933
OPU-113	*Staphylococcus* sp.	*Staphylococcus* sp. Bca53 / JQ977588
OPU-118	*Staphylococcus petrasii* subsp. *Croceilyticus*	*Staphylococcus petrasii* subsp. *croceilyticus* / AY953148

OPU-119	*Staphylococcus* sp.	*Staphylococcus capitis* subsp. *urealyticus* / AB009937
OPU-125	*Staphylococcus* sp.	*Staphylococcus sciuri* / AJ421446
OPU-127	*Jeotgalicoccus* sp.	*Jeotgalicoccus aerolatus* / GU295939
OPU-136	*Bacilli* sp.	uncultured bacterium / AM277404
OPU-137	*Bacilli* sp.	uncultured bacterium / JQ189451
OPU-149	*Bacilli* sp.	uncultured bacterium / EU475397
OPU-151	*Bacilli* sp.	uncultured bacterium / JQ191005
OPU-154	*Merdibacter* sp.	*Eubacterium* sp. Marseille-P3254 / FTLC01000013
OPU-155	*Erysipelotrichaceae* sp.	*Clostridiales bacterium* 60-7e / HQ452859
OPU-157	*Erysipelatoclostridium* sp.	*Massiliomicrobiota timonensis* / LN998062
OPU-159	*Erysipelatoclostridium spiroforme*	*[Clostridium] spiroforme* / X75908
OPU-170	*Lachnospirales* sp.	uncultured organism / HQ789378
OPU-174	*Lachnospirales* sp.	*Clostridium* sp. Marseille-P3244 / FQLT01000002
OPU-199	*Lachnospiraceae* sp.	uncultured bacterium / DQ456402
OPU-206	*Eisenbergiella* sp.	uncultured organism / HQ807825
OPU-250	*Oscillospiraceae* sp.	uncultured bacterium adhufec311 / AF132261
OPU-372	*Brachybacterium paraconglomeratum*	*Brachybacterium paraconglomeratum* / AJ415377
OPU-382	*Corynebacterium* sp.	*Corynebacterium stationis* / FJ172667
OPU-394	*Corynebacterium* sp.	uncultured organism / HQ753557
OPU-403	*Lawsonella* sp.	*Lawsonella clevelandensis* / CP009312
OPU-410	*Cutibacterium* sp.	uncultured bacterium / KF070955
OPU-411	*Cutibacterium granulosum*	*Cutibacterium granulosum* / AJ003057
OPU-430	*Gordonibacter* sp.	*Gordonibacter faecihominis* / KF785806
OPU-437	*Shigella dysenteriae* (*E. coli*)	*Shigella dysenteriae* (*E. coli*) / X96966
OPU-442	*Enterobacter* sp.	*Enterobacter cloacae* / KY855391
OPU-447	*Gallibacterium* sp.	*Gallibacterium anatis* / AF228001
OPU-463	*Massilia* sp.	*Massilia norwichensis* / HG798294
OPU-474	*Curvibacter gracilis*	*Curvibacter gracilis* / AB109889
OPU-495	*Sphingomonas* sp.	*Sphingomonas* sp. PL1 / JN849372
OPU-509	*Bacteroides fragilis* NCTC 9343	*Bacteroides fragilis* NCTC 9343 / CR626927
OPU-518	*Flavobacterium* sp.	*Flavobacterium chungbukense* / HM627539

Figure S3: Dendrogram analysis of tracheal samples. In the top panel, analysis between housing at week 1 (a), week 3 (b), and week 6 (c). In the bottom panel, analysis over time of sampling on BH-I (d), BH-II (e), and AF (f). Bray-Curtis distance method and Ward's clustering algorithm. AF: experimental animal facility, BH: broiler house. W1: week 1, W3: week 3, W6: week 6.

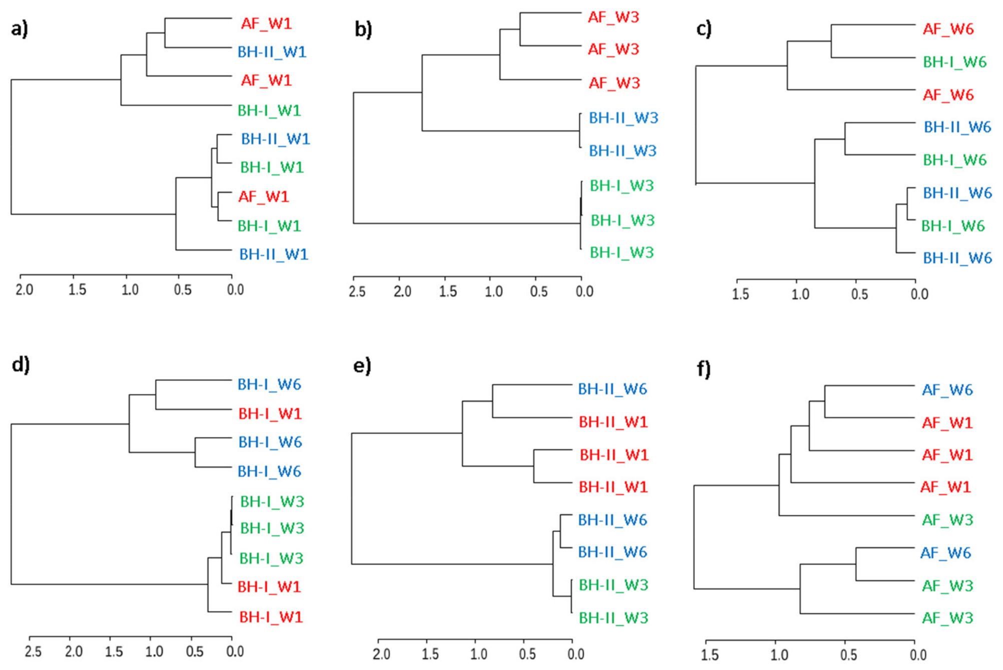

Figure S4: Beta-diversity analysis of environmental air and tracheal samples. PCoA of environmental air and tracheal samples by the week of sampling and housing using Bray-Curtis beta diversity measures. Statistics: PERMANOVA. In the top panel, analysis between air and trachea of housing—AF, BH-I and BH-II—at week 1 (a), week 3 (b), and week 6 (c). In the bottom panel, analysis between air and trachea through time of sampling—week 1, week 3 and week 6—on BH-I (d), BH-II (e), and AF (f). AF: experimental animal facility, BH: broiler house.

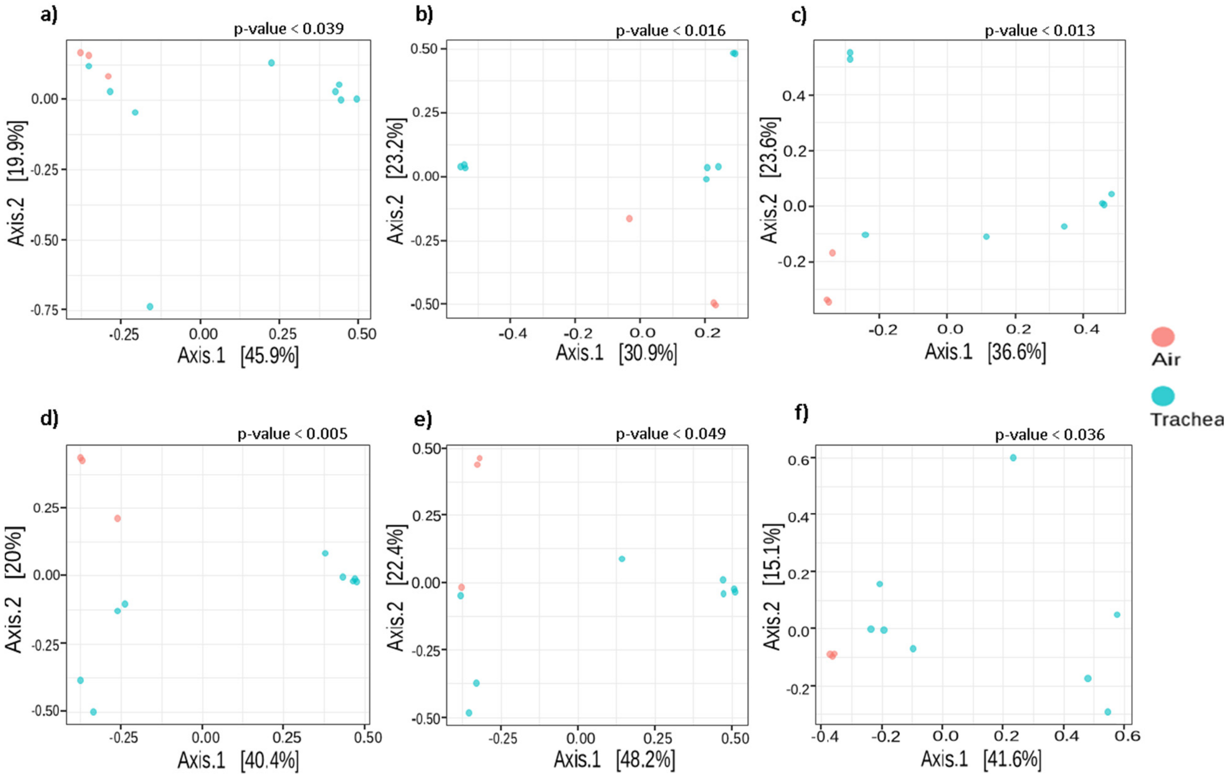

Figure S5: Sampling of 1000 liters of air in PCA plates with filter paper. The agar plates were incubated for 16 hours at 37 °C after removal of the filter paper. AF: experimental animal facility, BH: broiler house.

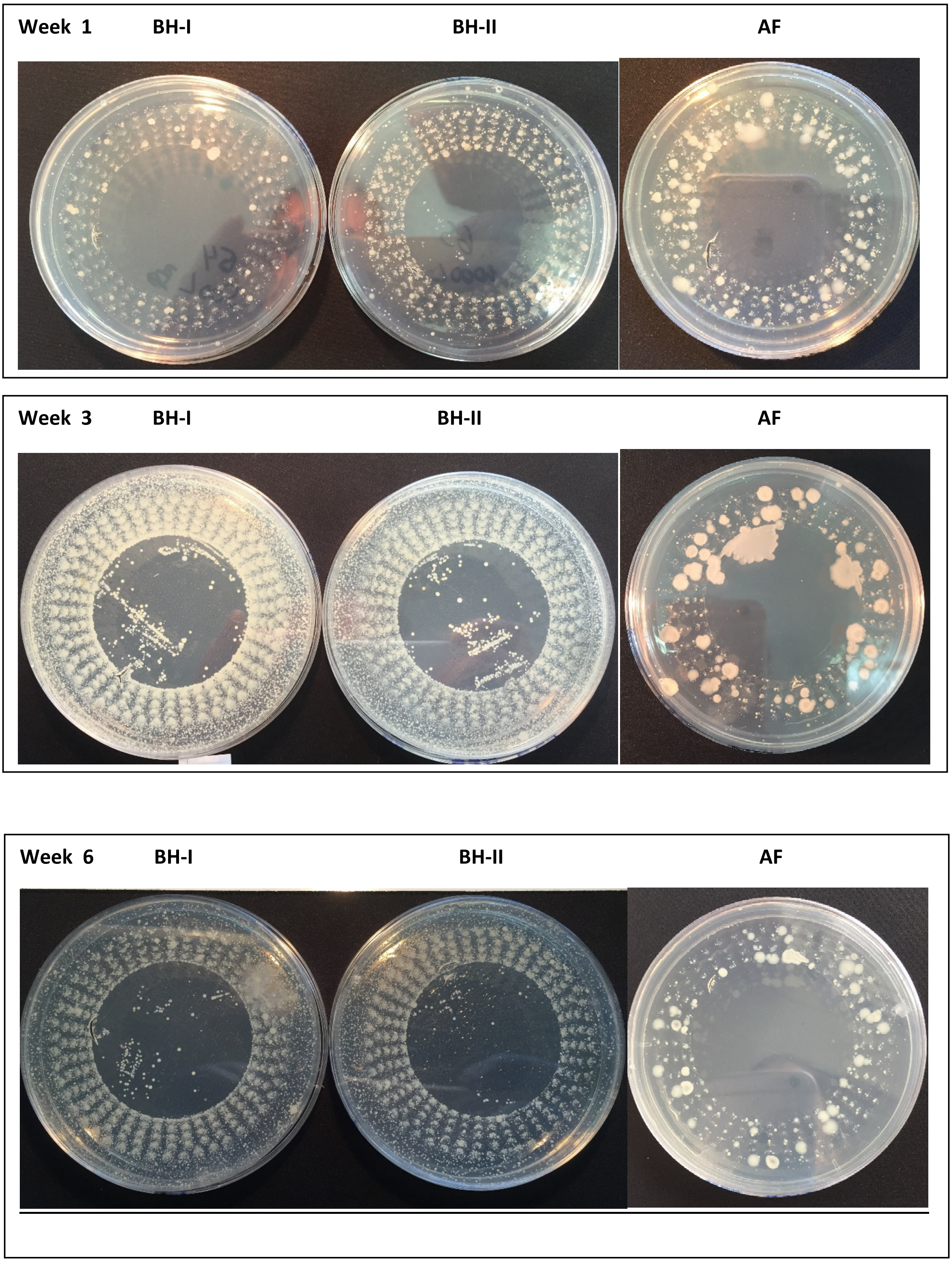

Figure S6. DNA electrophoresis of tracheal samples. Presence of 16S amplicon evaluated by DNA electrophoresis using primers 27F (5'- AGA GTT TGA TCC TGG CTC AG -3 ') and 1492R (5'- CGG TTA CCT TGT TAC GAC TT -3') in the amplification process. AF: experimental animal facility, BH: broiler house. W1: week 1, W3: week 3, W6: week 6. Lane 1, 2, 3, 4: negative controls. Lane 5: positive control. Asterisk (*): missing lane.

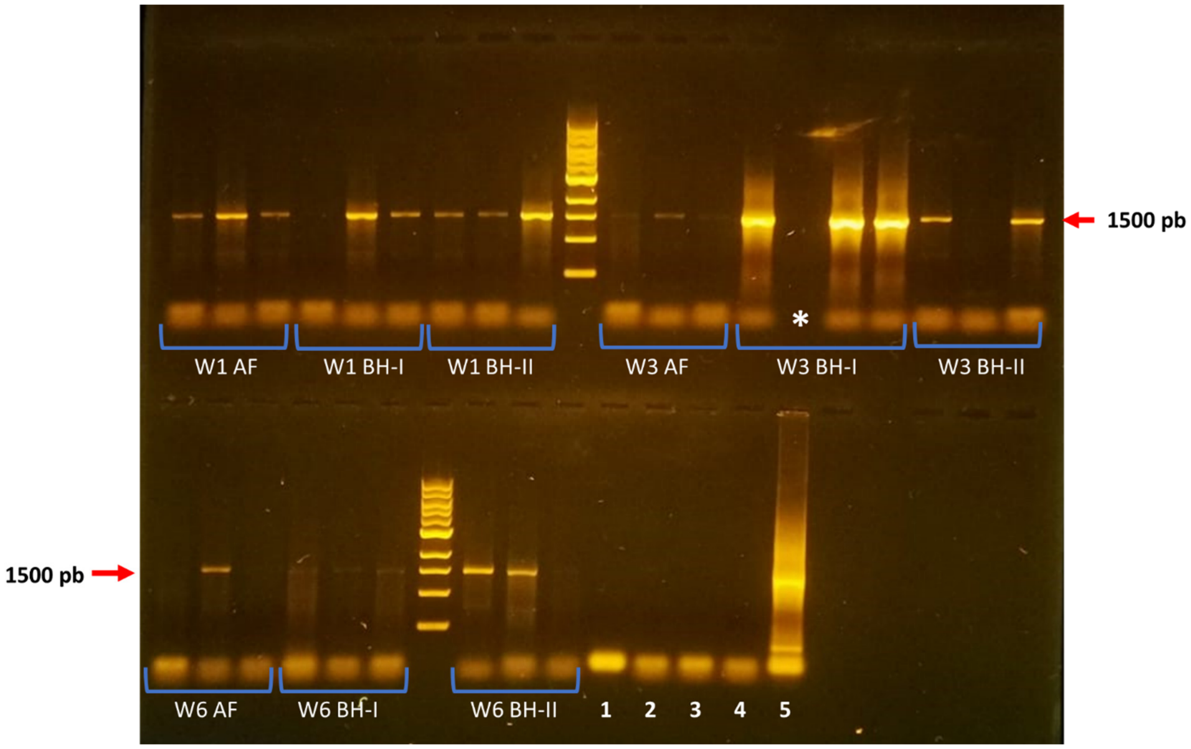

Supplement: Supplementary file 1 [file microorganisms-10-00878-s001.zip › microorganisms-1678894-supplementary.pdf]
